# Lysosomal Cathepsin A Plays a Significant Role in the Processing of Endogenous Bioactive Peptides

**DOI:** 10.3389/fmolb.2016.00068

**Published:** 2016-10-25

**Authors:** Zehra Kevser Timur, Secil Akyildiz Demir, Volkan Seyrantepe

**Affiliations:** Izmir Institute of Technology, Molecular Biology and GeneticsIzmir, Turkey

**Keywords:** Cathepsin A, bioactive peptid, mouse, lysosome, regulation

## Abstract

Lysosomal serine carboxypeptidase Cathepsin A (CTSA) is a multifunctional enzyme with distinct protective and catalytic function. CTSA present in the lysosomal multienzyme complex to facilitate the correct lysosomal routing, stability and activation of with beta–galactosidase and alpha-neuraminidase. Beside CTSA has role in inactivation of bioactive peptides including bradykinin, substances P, oxytocin, angiotensin I and endothelin-I by cleavage of 1 or 2 amino acid(s) from C-terminal ends. In this study, we aimed to elucidate the regulatory role of CTSA on bioactive peptides in knock-in mice model of *CTSA*^*S190A*^. We investigated the level of bradykinin, substances P, oxytocin, angiotensin I and endothelin-I in the kidney, liver, lung, brain and serum from *CTSA*^*S190A*^ mouse model at 3- and 6-months of age. Our results suggest CTSA selectively contributes to processing of bioactive peptides in different tissues from *CTSA*^*S190A*^ mice compared to age matched WT mice.

## Introduction

Lysosomal CathepsinA (CTSA), belonging to serine proteases family, is a multifunctional glycoprotein with three distinctive hydrolytic activities for deamidase, esterase and carboxypeptidase. It also makes a complex with the two glycosidases, β-galactosidase (β-gal) and α–neuraminidase (Neu1) to protect them against proteolytic degradation in lysosome (reviewed in Bonten et al., 2014). CTSA is necessary for oligomerization and catalytic activation of Neu1 (Itoh et al., 2000). Molecular defect of the CTSA cause autosomal recessively inherited rare lysosomal storage disease; galactosialidosis (D'azzo et al., 1982; Caciotti et al., 2013).

CTSA is widely distributed but differentially expressed in human tissues with the highest expression in the distal and collecting tubular cells of kidney, epithelial cells of lung, liver and large neurons of brain (Satake et al., 1994; Sohma et al., 1999). In mice, the highest level expression of CTSA is observed in kidney, brain, liver and placenta (Galjart et al., 1990). Secreted to the blood plasma by platelets and lymphocytes, CTSA very likely plays an extralysosomal (cell membrane) and/or extracellular regulatory role for a variety of bioactive peptide hydrolyzing them (Jackman et al., 1990).

CTSA is a member of α/β hydrolase fold family and has significant sequence homology in a conserved active side with two yeast enzymes, the KEX1 gene product and carboxypeptidase Y and wheat serine carboxypeptidase suggested the involvement of CTSA in different proteolytic functions in different cellular locations (Galjart et al., 1988; Hiraiwa, 1999). Several studies have shown that *in vitro* CTSA hydrolyze short regulatory bioactive short peptides including bradykinin, substances P, oxytocin, angiotensin I and endothelin-I (Jackman et al., 1992, 1993; Skidgel and Erdös, 1998). However, the physiological functions of CTSA outside the lysosome have not been explored fully. To elucidate this function, previously we have generated a knock-in mice model of *CTSA*^*S190A*^ by replacing the nucleophile of the CTSA active site, Serine at 190 with Alanine. While the mutant CTSA protein retained its ability to form a complex with β-gal and Neu1, it has abolished enzymatic activity. *CTSA*^*S190A*^ knock-in mice with inactive CTSA had a significantly induced level of bioactive short peptide endothelin-I resulting increased arterial blood pressure (Seyrantepe et al., 2008). In this study, we aimed to clarify significance of lysosomal CTSA in the regulation of several endogenous bioactive peptides including bradykinin, substances P, oxytocin, angiotensin I as well as endothelin-I in different tissues from *CTSA*^*S190A*^ mice model at two different age groups.

## Materials and methods

### Animals

*CTSA*^*S190A*^ knock-in mice model were previously generated by Dr. Volkan Seyrantepe in Montreal, Canada and donated by Dr. Alexey V. Pshezhetsky (Centre Hospitaliere Universitaire Sainte-Justine, University of Montreal, Montreal, Quebec, Canada). *CTSA*^*S190A*^ knock-in male mice was used and breed with C57/BL6 females for at least 10 generation to establish mice colony. All mice were bred and maintained in the Turkish Council on Animal Care (TCAC) accredited animal facility of Izmir Institute of Technology according to the TCAC guidelines. Mice were housed under constant room temperature and humidity on a 12 h light:dark cycle with *ad libitum* access to food and water. The animal care and the use in the experiments were granted by the Animal Care and Use Committee of Ege University, Izmir, Turkey.

### Sample preparation

3- and 6-months-old *CTSA*^*S190A*^ and wild-type (WT) littermate male mice were analyzed (*n* = 3). Blood samples were collected in a centrifuge tube (stabilized with EDTA) after the cardiac puncture under anesthesia. Blood was centrifuged at 4°C; 1600 g for 15 min and serum was stored at −80°C. Kidney, liver, lung and brain were taken, snap freezed with liquid nitrogen and kept in −80°C up to 30 days.

### Peptide isolation

For extraction of peptides, 350 mg of kidney, 1 g of liver, 170 mg of lung and 360 mg of brain from *CTSA*^*S190A*^ and WT male mice were homogenized in 5 ml/g cold lysis buffer (10 mM Tris, pH 7.4) using homogenizer (IKA T10 Basic Ultra-Turra-fold). After centrifugation of homogenate (1600 g for 15 min at 4°C), Aprotinin (RK-APRO) was added to all samples including serum at 1/10 (v/v) ratio. Peptides were purified with SEP-Pak C18 column (Phoenix Strata) from homogenized tissues and serum of mice. Eluted samples were first lyophilized (Labconco-Freezone Lyophilizer) and then pellet were re-suspended with ELISA 1-fold Assay Buffer.

### ELISA assays

Peptides from kidney, liver, lung, brain and serum were analyzed using Enzyme Immuno Assay kit (Phoenix Pharmaceutical—Bradykinin, Substances P, Oxytocin, Angiotensin I and Endothelin-I) according to manufacturer's instruction.

### Statistical analysis

Collected data correspond to means of 3 different animals with same sex age and genotype was analyzed using one-way ANOVA. Data were considered significant if *P* < 0.05.

## Results

In the concept of this study, we determined the level of five different endogenous bioactive peptides (bradykinin, substances P, oxytocin, angiotensin I and endothelin-I) in kidney, liver, lung, brain and serum from *CTSA*^*S190A*^ and *WT* male mice at 3- and 6-months-old in order to reveal the importance of lysosomal CTSA on the regulation of bioactive peptides *in vivo*.

### Kidney

**Figure 1:**
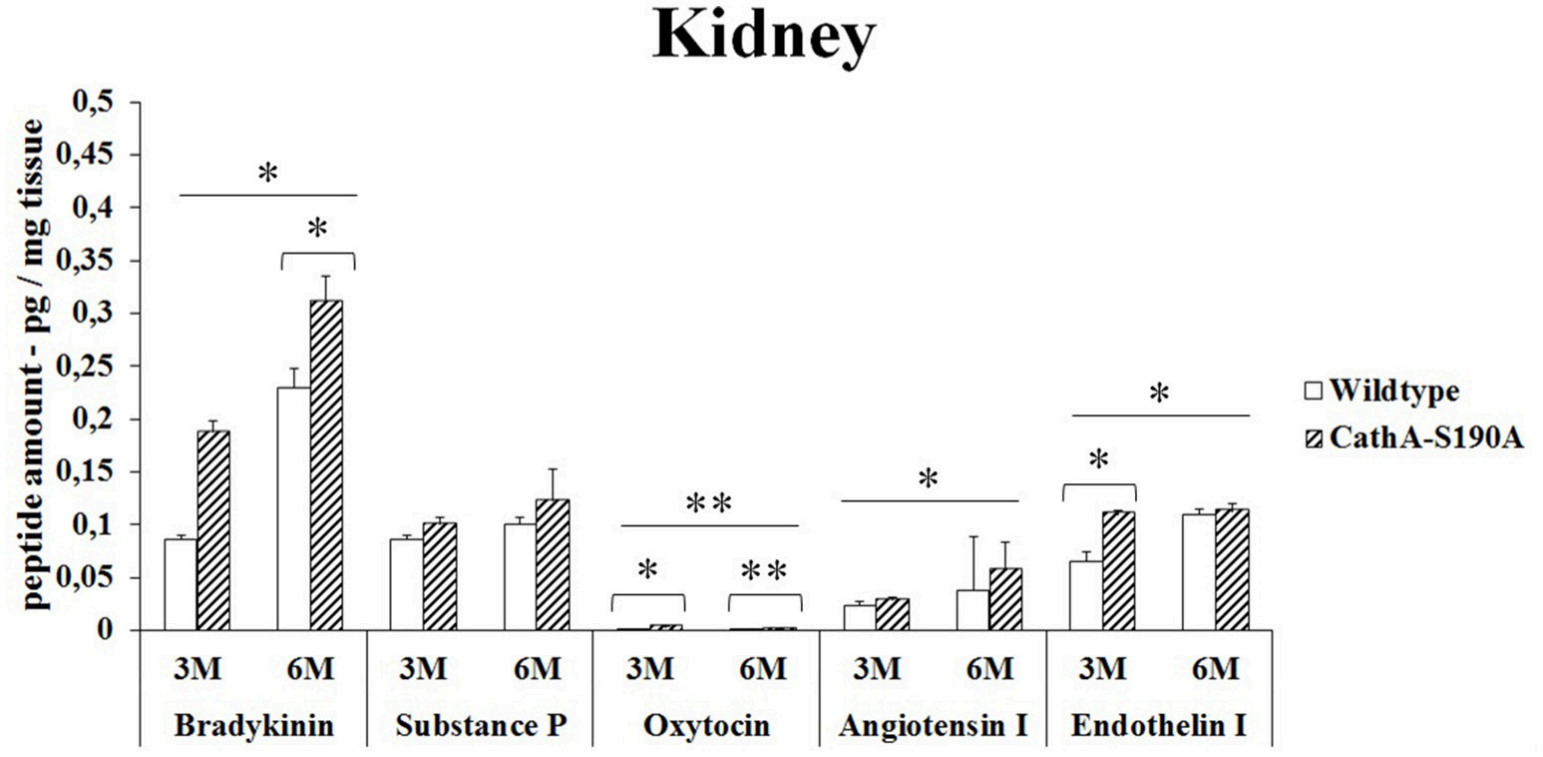
**The levels of bradykinin, substance P, oxytocin, angiotensin I and endothelin-I in the kidney of 3-month-old (3M) and 6-month-old (6M) CathA^**S190A**^ mice and WT littermate mice**. Data show mean± SE of measurements in three male mice. Significant levels of Student's *t*-tests are shown below the boxplots. The symbol “ ____ ” indicates significant levels of bradykinin, substance P, angiotensin I and endothelin I between 3- and 6-month-old CathA^S190A^ mice using the one-way ANOVA (^*^p < 0.05 and ^**^p < 0.025).

*CTSA*^*S190A*^ male mice at the age of 3 months had elevated levels of bradykinin (2.2-fold), oxytocin (2.3-fold), angiotensin-I (1.2-fold) and endothelin-I (1.6-fold) compared to WT mice in kidney. However, no differences were detected in the levels of substances P in the same tissue from age matching *CTSA*^*S190A*^ and *WT* male mice (Figure 1). In 6 month old *CTSA*^*S190A*^ mice's kidney, the level of bradykinin, oxytocin and angiotensin-I were higher (1.3-fold, 1.2-fold and 1.5-fold, respectively) whereas substances P and endothelin-I levels were similar to age matching WT mice (Figure 1).

### Liver

**Figure 2:**
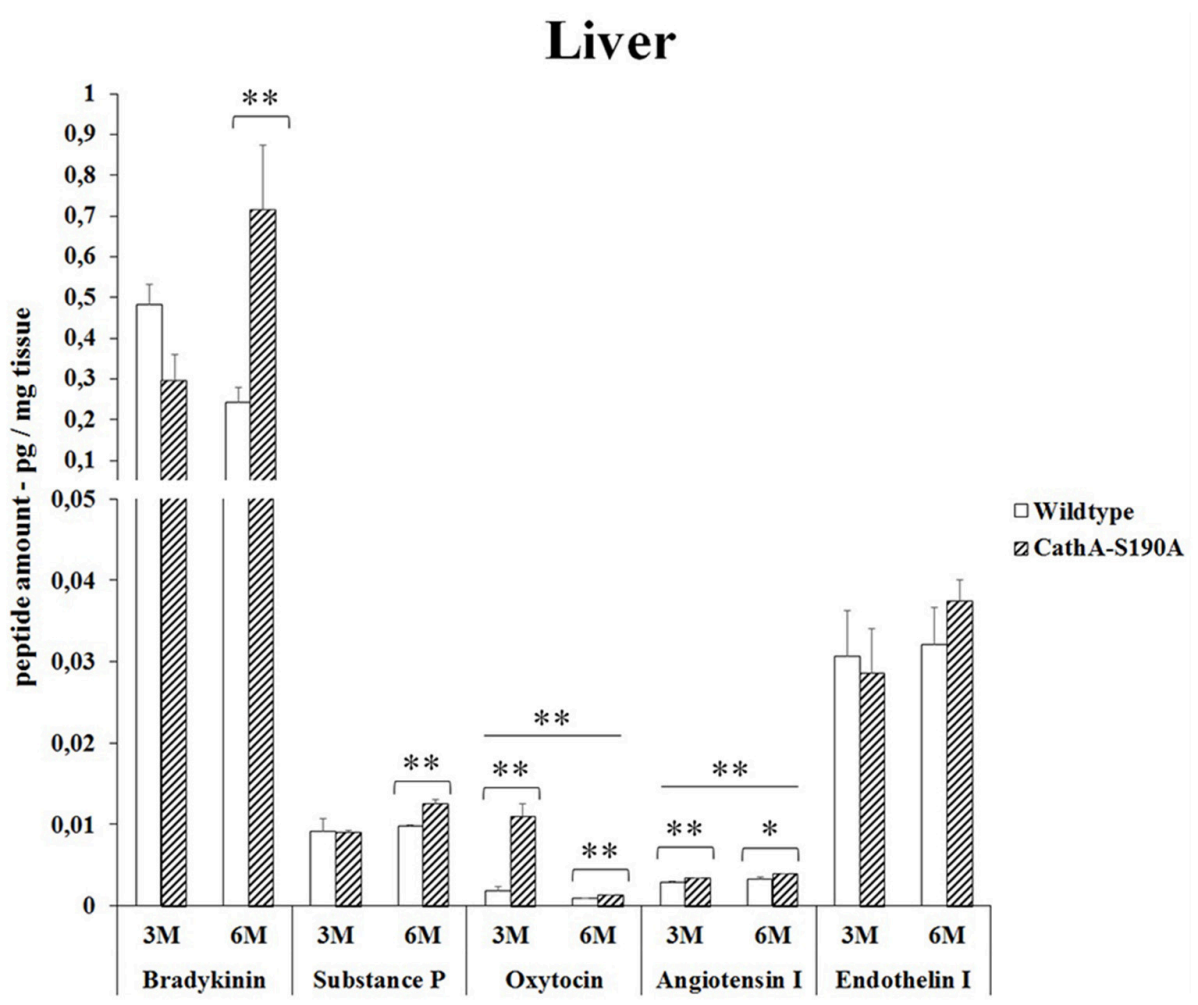
**The levels of bradykinin, substance P, oxytocin, angiotensin I and endothelin-I in the liver of 3-month-old (3M) and 6-month-old (6M) CathA^**S190A**^ mice and WT littermate mice**. Data show mean ± SE of measurements in three male mice. Significant levels of Student's *t*-tests are shown below the boxplots. The symbol “ ____ ” indicates significant level of oxytocin and angiotensin I between 3- and 6-month-old CathA^S190A^ mice using the one-way ANOVA (^*^p < 0.05 and ^**^p < 0.025).

3 months old *CTSA*^*S190A*^ male mice showed significant increase in oxytocin level (5.8 -fold) in liver while other peptides were similar to *WT*. However, *CTSA*^*S190A*^ male mice at the age of 6 month, had higher levels of bradykinin (2.9 -fold) and substances P (1.3-fold) in same tissue compared to *WT* (Figure 2). Lower oxytocin level was detected in older *CTSA*^*S190A*^ however it was higher (1.4-fold) as compared to WT mice.

### Lung

**Figure 3:**
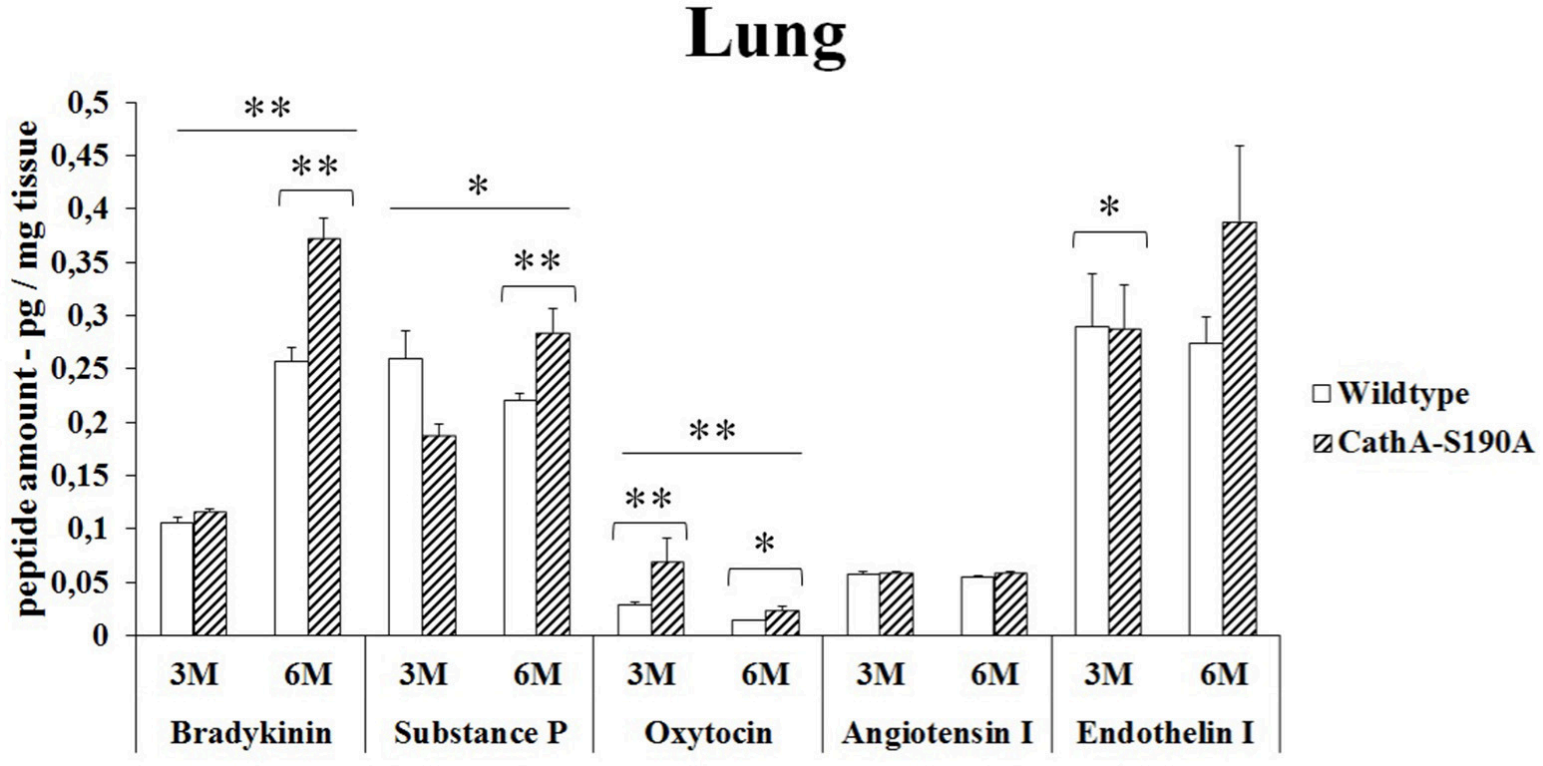
**The levels of bradykinin, substance P, oxytocin, angiotensin I and endothelin-I in the lung of 3-month-old (3M) and 6-month-old (6M) CathA^**S190A**^ and WT littermate mice**. Data show mean ± SE of measurements in three male mice. Significant levels of Student's *t*-tests are shown below the boxplots. The symbol “ ____ ” indicates significant levels of bradykinin, substance P and oxytocin between 3- and 6-month-old CathA^S190A^ mice using the one-way ANOVA (^*^p < 0.05 and ^**^p < 0.025).

Bradykinin (1.5-fold), substances P (1.3-fold), oxytocin (1.6-fold) and endothelin-I (1.4-fold) levels were significantly higher in the lung tissue from 6 months old *CTSA*^*S190A*^ male mice but not in younger mice. No difference was detected in the levels of Angiotensin I in the lung from 6 months old *CTSA*^*S190A*^ and *WT* male mice. Only oxytocin level in lung tissue from 3 months old *CTSA*^*S190A*^ male mice was higher (2.4-fold) compared to *WT* male mice (Figure 3).

### Brain

**Figure 4:**
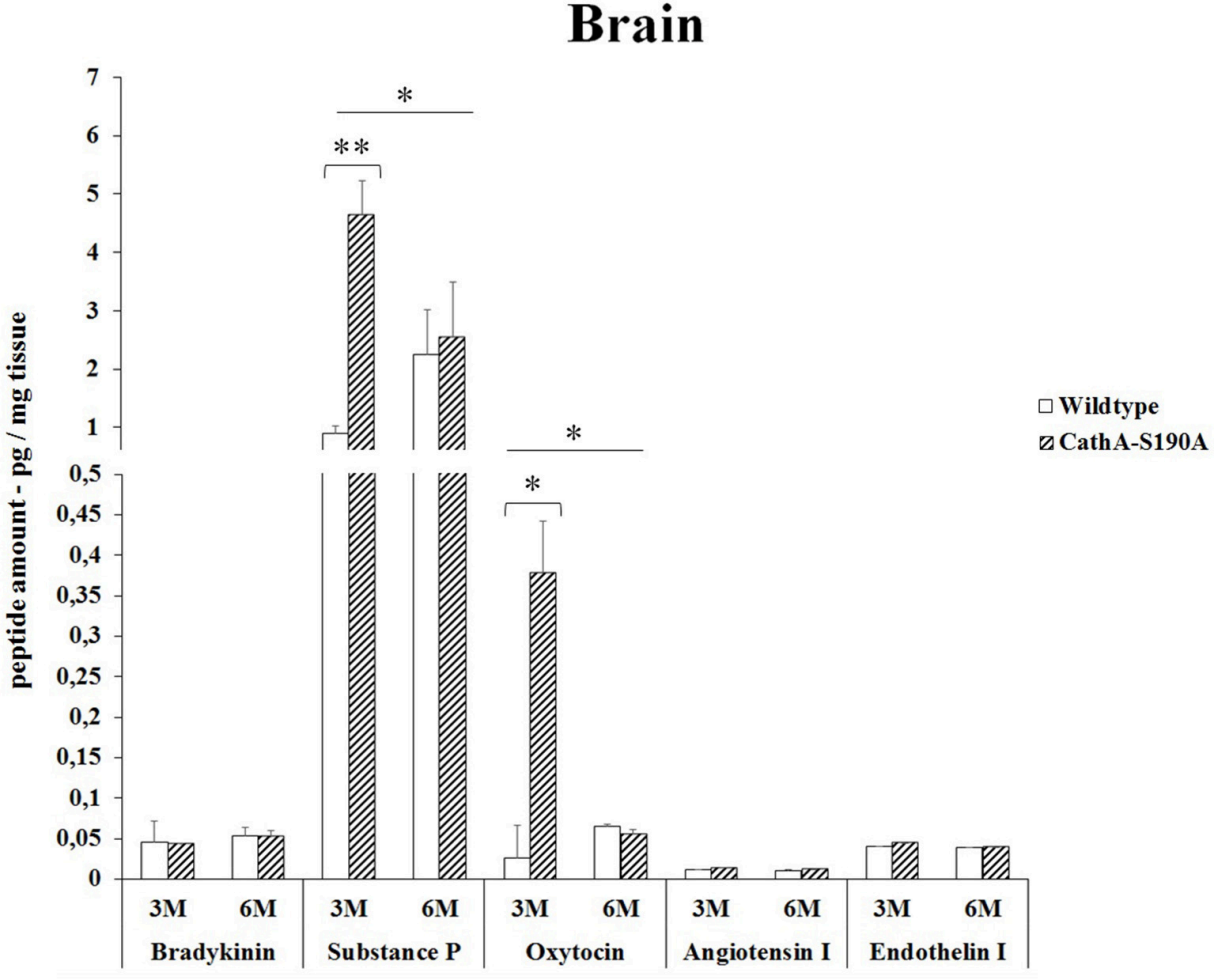
**The levels of bradykinin, substance P, oxytocin, angiotensin I and endothelin-I in the brain of 3-month-old (3M) and 6-month-old (6M) CathA^**S190A**^ and WT littermate mice**. Data show mean ± SE of measurements in three male mice. Significant levels of Student's *t*-tests are shown below the boxplots. The symbol “ ____ ” indicates significant level of substance P and oxytocin between 3- and 6-month-old CathA^S190A^ mice using the one-way ANOVA (^*^p < 0.05 and ^**^p < 0.025).

In the brain, the analysis of the bioactive peptides showed that oxytocin (14.2-fold) and substances P (5.2-fold) level was significantly higher in only 3 months old *CTSA*^*S190A*^ mice compared to *WT* mice. However, no accumulations of other peptides were detected (Figure 4).

### Serum

**Figure 5:**
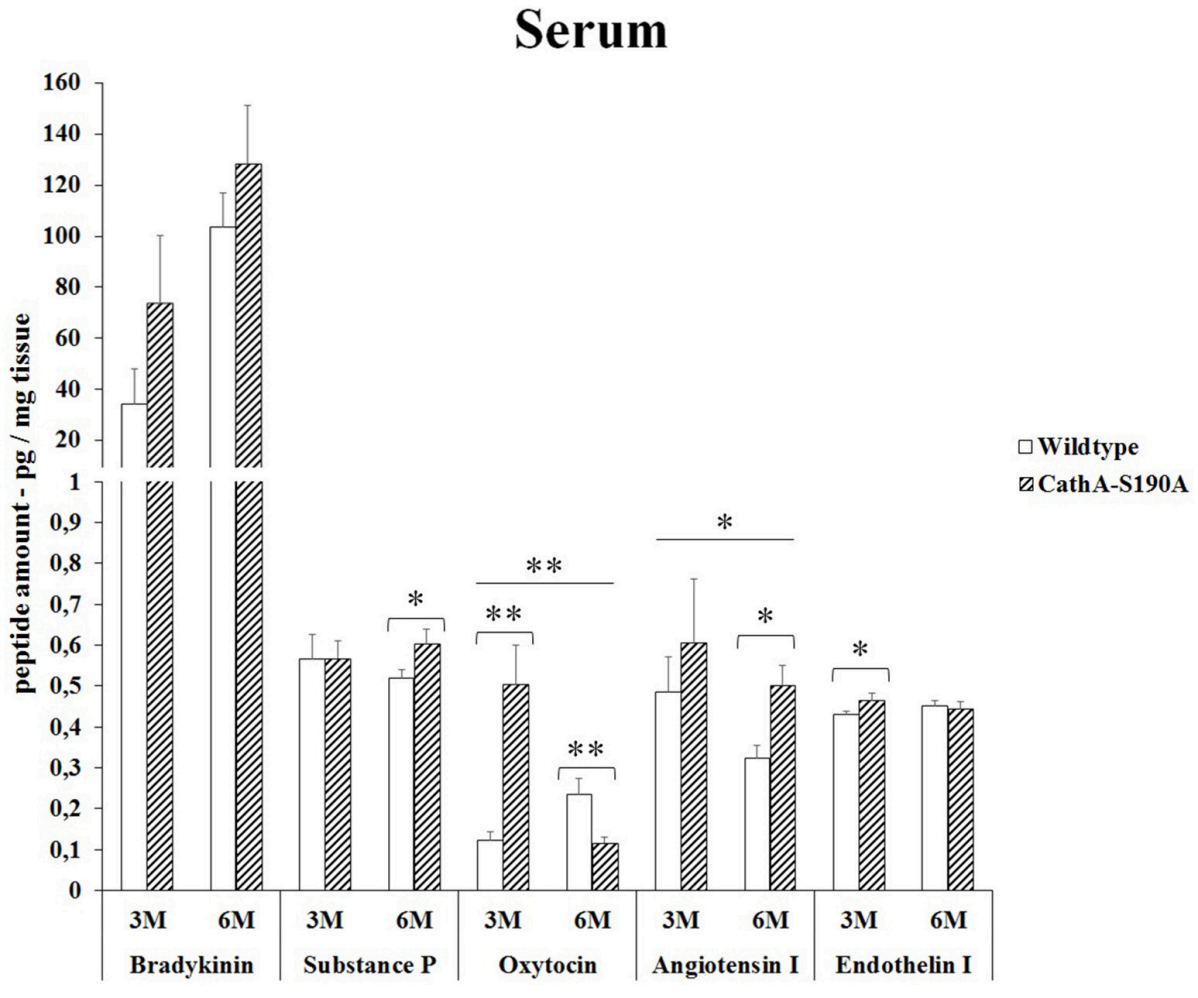
**The levels of bradykinin, substance P, oxytocin, angiotensin I and endothelin-I in the serum of 3-month-old (3M) and 6-month-old (6M) CathA^**S190A**^ and WT littermate mice**. Data show mean ± SE of measurements in three male mice. Significant levels of Student's *t*-tests are shown below the boxplots. The symbol “ ____ ” indicates significant level of oxytocin and angiotensin I between 3-and 6-month-old CathA^S190A^ mice using the one-way ANOVA (^*^p < 0.05 and ^**^p < 0.025).

We detected significantly higher level of bradykinin in serum from 3 months old (2.4-fold) as well as 6 months old (1.2-fold) *CTSA*^*S190A*^ male mice compared to age-matched *WT* mice. The level of oxytocin in serum of *CTSA*^*S190A*^ mice was higher (4.1-fold) in 3 months old but not in the older mice. On the other hand, the levels angiotensin-I were increased in both 3 and 6 months old *CTSA*^*S190A*^ mice's serum as 1.3-fold and 1.6-fold, respectively (Figure 5).

## Discussion

CTSA is a lysosomal protein with dual function: protective and catalytic. CTSA associates with β-galactosidase and α–neuraminidase forming a multi-enzyme complex to protect these two enzymes against lysosomal degradation. Independent from its protective function, CTSA has carboxpeptidase activity at an acidic pH optimum between pH 4.5 and 5.5 as well as deamidase and esterase activities at neutral pH which leads the deamination of peptides (Hiraiwa, 1999).

The carboxyl-terminal end of many physiologically active peptides is blocked by an amide bond (−CONH_2_) and these form of the peptide are less susceptible to degradation. Several *in vitro* studies showed that CTSA is involved in activation or inactivation of these peptides by removing last one or two amino acid residues (Skidgel and Erdös, 1998). It has been shown that CTSA hydrolyzes a variety of bioactive peptides including substances P (Jackman et al., 1990). Report on the different percentage of relative carboxypeptidase activity against bioactive peptides such as endothelin I (100%), angiotensin I (9.5%), bradykinin (6.1%), oxytocin (4%), substances P (3.8%) suggests that CTSA may play a role in regulation of bioactive peptide functions (Hiraiwa, 1999).

Previously, it has been demostrated that expression level of CTSA enzyme has a tissue specific pattern in mice (Galjart et al., 1990). Although, kidney has the highest, relatively high levels of expression in brain, liver and lung implicates that CTSA may have different level of contribution to the hydrolysis of endogeneous bioactive peptides in different tissues. To elucidate the *in vivo* significance of lysosomal CTSA in the regulation of several endogenous bioactive peptides, we analyzed the levels of bradykinin, substances P, oxytocin, angiotensin I as well as endothelin-I in different tissues from knock-in *CTSA*^*S190A*^ male mice at two different age groups.

Endothelin-I is a peptide composed of twenty-one amino acids. It is produced mainly in endothelial cells, vascular smooth muscle cells and a lesser extent by astrocytes, neurons, sertoli cells and hepatocytes. Endothelin-1 functions as vasoconstrictor and affects the salt-water equilibrium (Barnes and Turner, 1997). Endothelin-I is the well-known substrate for CTSA (Jackman et al., 1992). Previously we have reported that *CTSA*^*S190A*^ mice have an increased level of endothelin-I only in lungs (Seyrantepe et al., 2008). Our current data showed that not only in lungs but also in kidney, there is increased level of endothelin-I in *CTSA*^*S190A*^ mice at 6 and 3 months old respectively. Although, the markedly increased endothelin-1-specific immunoreactivity has been demonstrated in CTSA deficient galactosialidosis patient's brain, we did not observe any difference neither in brain nor in liver from *CTSA*^*S190A*^ mice using ELISA (Itoh et al., 2000). We also speculate that higher expression of other carboxypeptidases such as Scpep1 may contribute modulating the catabolic proteolysis of Endothelin-I in these tissues (Pshezhetsky and Hinek, 2009; Pan et al., 2014).

Bradykinin is a physiologically and pharmacologically active peptide consists of nine amino acids known to influence the inflammatory process by affecting various tissue reactions including vasodilation, permeability of vessels and coagulation (Maurer et al., 2011). As a neuropeptide, bradykinin in brain chemotactically attracts glioma cells to blood vessels causing their migration and significantly changes the physiological properties of these cells *in vivo* (Montana and Sontheimer, 2011). We report for the first time that significantly higher levels of bradykinin in kidney (Figure 1), liver (Figure 2), lung (Figure 3) and serum (Figure 5) of the *CTSA*^*S190A*^ mice compared to *WT* mice which indicates CTSA has important role on inactivation of endogenous bradykinin peptide *in vivo*. Interestingly, the comparisons of bradykinin levels in brain from 3 and 6 months old *CTSA*^*S190A*^ and their WT siblings measured by ELISA were not conslusive. Therefore, we suggest that quantitation of bradykinin in brain using 2D-LC-MS/MS assays may be uniquely advantageous for elucidating physiological and biological events.

Substance P consists of eleven amino acids which is released from both the central and peripheral endings of neurons and functions as a neurotransmitter which is involved in vasodilation (reviewed in Harrison and Geppetti, 2001). Substances P plays role in inflammation through astrocyte and leukocyte activation at the site of injury (Lorente et al., 2015). A number of enzymes are required in the metabolism of substances P, including: neutral endopeptidase (NEP), angiotensin-converting enzyme (ACE), substance-P-degrading enzyme, cathepsin-D and cathepsin-E. In our study, among the studied tissues, a significant accumulation of substances P (5-fold) was observed in the brain of *CTSA*^*S190A*^ mice (Figure 4). This accumulation in the absence of active CTSA shows that beside other enzymes CTSA is one of the functional enzymes that are involved in the metabolism of substances P, especially in mouse brain.

The role of CTSA in conversion of angiotensin-I to angiotensin-II suggests that CTSA may be an important player of the renin-angiotensin system regulating blood pressure and water balance (Miller et al., 1988). In the classic renin-angiotensin system enzymatic cascade angiotensinogen is cleaved by renin and converted to angiotensin I which is further processed by angiotensin converting enzyme (ACE) to form angiotensin II (Lavoie et al., 2004). Similar to ACE, CTSA is more likely to be involved in the *in vivo* conversion of angiotensin I into angiotensin II in the human heart (Jackman et al., 2002). In our results it was showed that elevated levels of angiotensin-I in kidney (Figure 1) and serum (Figure 5) but not in liver (Figure 2), lung (Figure 3) and brain (Figure 4) of *CTSA*^*S190A*^ mice. Since Angiotensin-I is an intermediary peptide found most abundant as inactive form in blood (Lavoie et al., 2004), it is not surprising that we were able to detect the increased level of angiotensin I in blood as well as kidney where the cleavage of angiotensinogen takes place. Our results clearly implicate that CTSA is important member of renin-angiotensin system, however, more precise study including *in situ* analysis showing the expression level of CTSA and Angiotensin-I in particular tissues such as renal proximal tubules is required.

Oxytocin is a mammalian, 9-amino acid cyclic peptide which is synthesized in biologically active form in the paraventricular and supra-optic nuclei of the hypothalamus and is released into the central nervous system as well as the bloodstream (reviewed in Lee et al., 2009). In addition to brain, oxytocin and oxytocin receptors are found in several peripheral organs including heart, kidney, pancreas, breast, thymus, non-pregnant uterus, bowel and testis of the rat and human (Vargas-Martínez et al., 2014). Although, the highest densities of oxytocin receptors were observed in infant rat brain especially in cortex and spinal cord, low or undetectable numbers of oxytocin receptors were reported in the adult rat brain. It has been shown that aging decreases the number of oxytocin binding sites in the other part of brain (Arsenijevic et al., 1995). Besides its best known roles in parturition and lactation, oxytocin also plays important regulatory role in variety of physiological conditions such as social memory and attachment, sex and maternal behavior, and human bonding and trust (Gimpl and Fahrenholz, 2001; Lee et al., 2009). Oxytocin and oxytocin receptor expression is effected by steroid hormones and gender and it is usually higher in females (Lee et al., 2009). Therefore, in our research only male mice were investigated to detect the regulatory function of CTSA on cleavage off oxytocin peptide *in vivo*, kidney, liver, lung, brain and serum. Our results showed that the levels of oxytocin peptide were significantly higher in kidney (2.3-fold), liver (5.8-fold), lung (2.4-fold), brain (14.2-fold) and serum (4.1-fold) of 3 months old *CTSA*^*S190A*^ mice compared to age matched *WT* mice respectively. Our data clearly suggests the role of CTSA on the cut off oxytocin in different tissues. The decrease in the oxytocin level in serum and other tissues of 6 months of *WT* and *CTSA*^*S190A*^ mice as compared to that of 3 months may be related to the decreased level of oxytocin receptors in the aging brain.

Over all, our data clearly indicates that CTSA contributes on the hydrolysis of bioactive peptides in different mouse tissues including kidney, liver, lung, brain and serum. However, we expect that further analysis of aging mice (12 months old or older) which either can be feed with high-salt diet or normal diet may provide more insights regarding CTSA catalytic activity on bioactive peptides and its physiological role outside of lysosomes. Additionally, high-throughput and selective 2D-LC-MS/MS bioanalytical methods rather than sensitive radioimmunoassay (RIA) or ELISA can be applied to validate the physiological role of this unique enzyme in the regulation of bioactive peptides as well as determination of its unknown substrates.

## Author contributions

All authors listed, have made substantial, direct and intellectual contribution to the work, and approved it for publication.

### Conflict of interest statement

The authors declare that the research was conducted in the absence of any commercial or financial relationships that could be construed as a potential conflict of interest.
